# Supplementary material for: Cardiac Development Long Non-Coding RNA (CARDEL) Is Activated during Human Heart Development and Contributes to Cardiac Specification and Homeostasis
Source: Cells. 2024 Jun 18;13(12):1050. doi: 10.3390/cells13121050 (PMC11201801; doi:10.3390/cells13121050)
Supplement: Supplementary file 1 [file cells-13-01050-s001.zip › Sup_material/Supplementary Methods.pdf]

Supplementary Methods
Pereira et al.

Nucleus/Cytoplasm Fractionation

The nucleus and cytoplasm fractionation protocol was adapted from SENICHKIN *et al.*, 2021 (doi:10.3390/cells10040852). Cells on day 10 of the cardiac differentiation protocol were lysed with hypotonic buffer (20 mM Tris-HCl; 10 mM KCl; 2 mM $MgCl2$; 0.1% NP-40) for precisely 3 minutes. Next, the cell extract was centrifuged at 1,000 xg for 5 minutes to precipitate the nuclear fraction. The supernatant was centrifuged again at 15,000 xg for 3 minutes to discard the remaining debris. RNA from each fraction was isolated using the Direct-zol RNA Purification Kit (Zymo Research). cDNA synthesis was performed using the ImProm-II™ Reverse Transcription System (Promega) with the same volume (1 µL) of RNA from each fraction. During synthesis, 5 pg of GFP RNA was added as a spike-in for each reaction. RT-qPCR reactions were performed using the GoTaq® qPCR Master Mix (Promega). Analyses were carried out using the QuantStudio™ 5 Real-Time PCR System. GFP detection was used as a normalization control.

Endoderm and Ectoderm differentiation

For endoderm differentiation, the cells were subjected to a protocol adapted from CARUSO *et al.,* 2023 (https://doi.org/10.1016/j.stemcr.2022.11.020). hESC and hiPSC cells were seeded at 5×10^4 cells/cm^2 in a 24-wells plate pre-coated with Geltrex hESC-qualified (Thermo Fisher Scientific) in StemFlex medium (Thermo Fisher Scientific) with 10 µM of Y27632 (Med Chem Express). After 24 hours, the medium was replaced to remove the ROCK inhibitor. On the following day (D0), the medium was change to RPMI 1640 (Gibco) supplemented with 1% l-

glutamine, 1% non-essential amino acids and 2% B-27 minus insulin (Gibco), the induction was performed by adding 3 μM of CHIR99021 (TOCRIS) and 50 ng/ml of Activin A (B&D systems) during the medium replacement. After 2 days (D2), the medium was replaced to the base medium containing only 50 ng/ml Activin A (B&D systems). On D4, cells were collected to RNA isolation.

For ectodermal differentiation, hESC and hiPSC cells were seeded at 2,5 x 10^4 cells/cm^2 in a 6-wells plate pre-coated with Geltrex hESC-qualified (Thermo Fisher Scientific) in StemFlex medium (Thermo Fisher Scientific) with 10 μM of Y27632 (Med Chem Express). The next day (D0), the medium was changed to Neural Induction Medium (Thermo Fisher Scientific). Following the manufacturer, the medium was changed on days 3, 5, and 7. On D8, cells were collected to RNA isolation.
