# Supplementary figures and images for: Cardiac Development Long Non-Coding RNA (CARDEL) Is Activated during Human Heart Development and Contributes to Cardiac Specification and Homeostasis
Source: Cells. 2024 Jun 18;13(12):1050. doi: 10.3390/cells13121050 (PMC11201801; doi:10.3390/cells13121050)

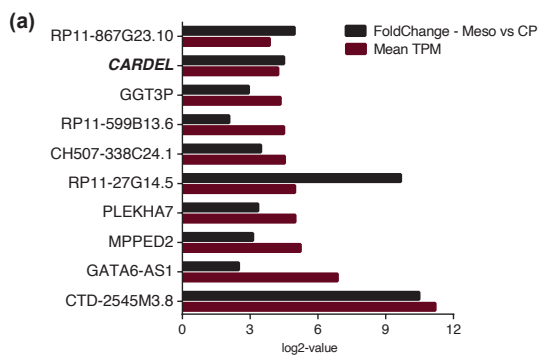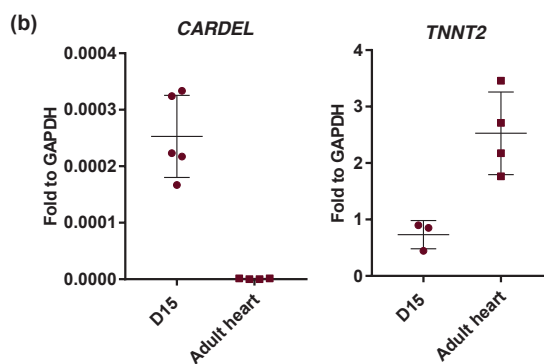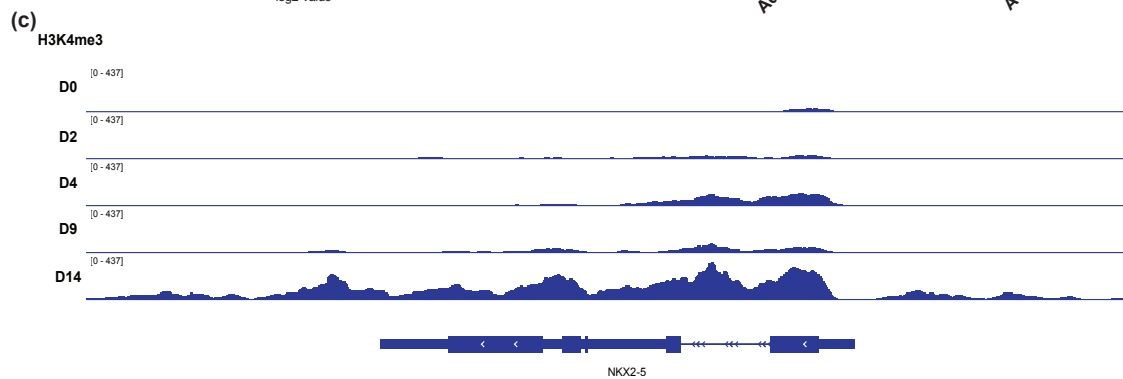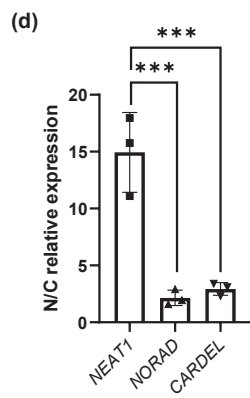

Supplement: Supplementary file 1 [file cells-13-01050-s001.zip › Sup_material/FigureS1.pdf]

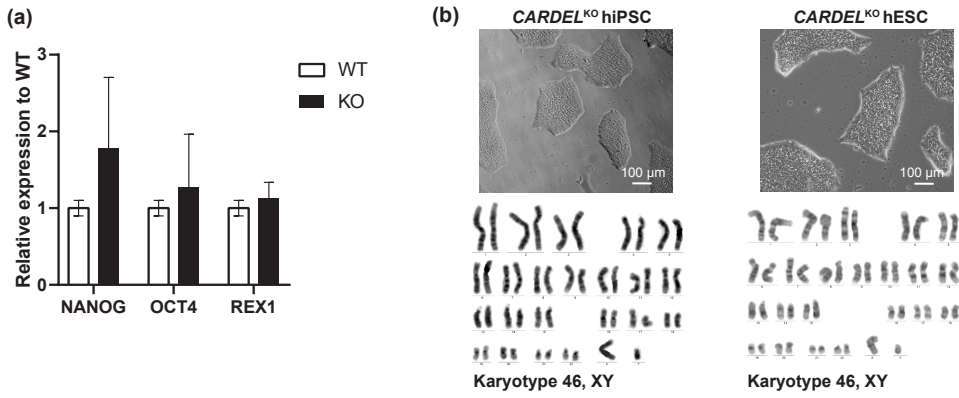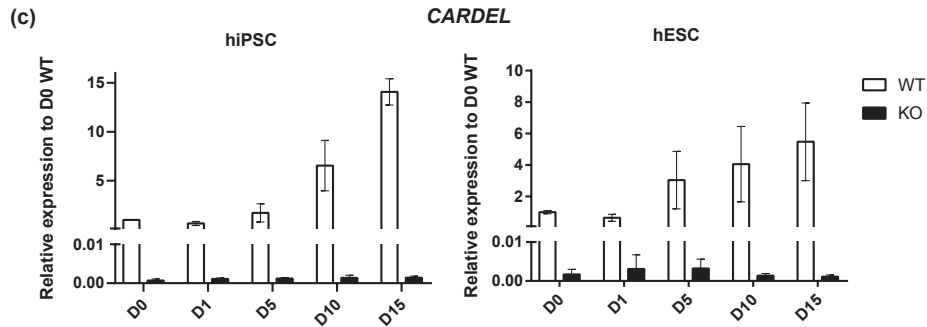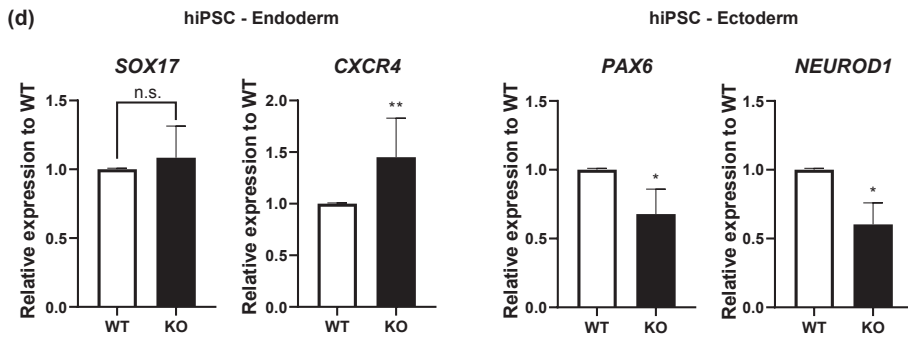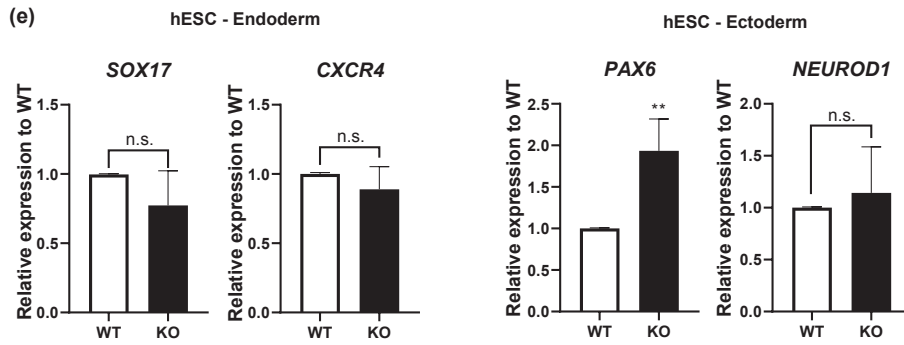

Supplement: Supplementary file 1 [file cells-13-01050-s001.zip › Sup_material/FigureS2.pdf]

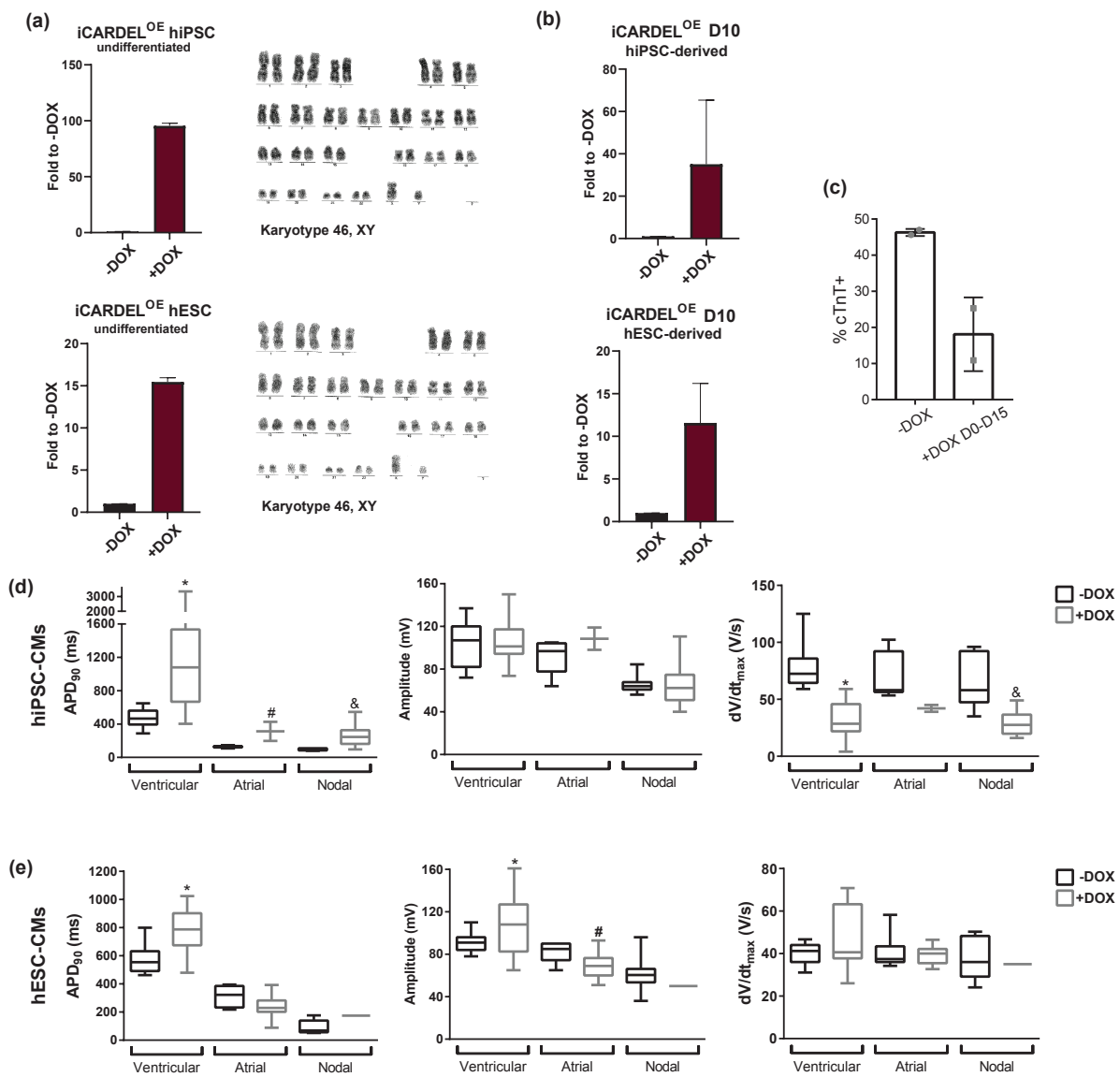

Supplement: Supplementary file 1 [file cells-13-01050-s001.zip › Sup_material/FigureS3.pdf]
